# Unsustainable Growth, Hyper-Competition, and Worth in Life Science Research: Narrowing Evaluative Repertoires in Doctoral and Postdoctoral Scientists’ Work and Lives

**DOI:** 10.1007/s11024-016-9292-y

**Published:** 2016-03-04

**Authors:** Maximilian Fochler, Ulrike Felt, Ruth Müller

**Affiliations:** Department of Science and Technology Studies, University of Vienna, Universitaetsstrasse 7, 1010 Vienna, Austria; Munich Center for Technology in Society, Technical University Munich (TUM), Munich, Germany

**Keywords:** Life sciences, Valuation, Impact factor, Growth, Competition, Academic socialization, Metrics, Evaluation, Early-stage researchers, Research careers, Postdoc

## Abstract

There is a crisis of valuation practices in the current academic life sciences, triggered by unsustainable growth and “hyper-competition.” Quantitative metrics in evaluating researchers are seen as replacing deeper considerations of the quality and novelty of work, as well as substantive care for the societal implications of research. Junior researchers are frequently mentioned as those most strongly affected by these dynamics. However, their own perceptions of these issues are much less frequently considered. This paper aims at contributing to a better understanding of the interplay between how research is valued and how young researchers learn to live, work and produce knowledge within academia. We thus analyze how PhD students and postdocs in the Austrian life sciences ascribe worth to people, objects and practices as they talk about their own present and future lives in research. We draw on literature from the field of valuation studies and its interest in how actors refer to different forms of valuation to account for their actions. We explore how young researchers are socialized into different valuation practices in different stages of their growing into science. Introducing the concept of “regimes of valuation” we show that PhD students relate to a wider evaluative repertoire while postdocs base their decisions on one dominant regime of valuing research. In conclusion, we discuss the implications of these findings for the epistemic and social development of the life sciences, and for other scientific fields.

A sense of crisis currently pervades the academic life sciences. In the US (Alberts et al. 2014) and in Europe (Schatz 2014), eminent senior scholars state that their field has deep epistemic and social problems because of growth and the resulting “hyper-competition.” Hyped as the leading sciences of the 21^st^ century, the life sciences have been very successful in attracting public money in recent decades. However, the almost explosive growth of third party-funded pre- and postdoctoral temporary job opportunities has not been matched by the number of new faculty positions (Stephan 2012). Nobel laureates and former Ivy League presidents warn that the resulting competition is “suppressing the creativity, cooperation, risk-taking, and original thinking required to make fundamental discoveries” (Alberts et al. 2014: 5774).

However, unsustainable growth is not the only dimension of this crisis; it is also a crisis of academic valuation practices. Along with his co-authors, the former editor-in-chief of *Science* magazine argues that “the inflated value given to publishing in a small number of so-called ‘high impact’ journals has put pressure on authors to rush into print, cut corners, exaggerate their findings, and overstate the significance of their work” (Alberts et al. 2014: 5774). Evaluating researchers by counting impact factors and using other quantitative metrics has seemingly replaced deeper considerations of the quality and novelty of their work, as well as adequate concern about the societal implications of their research (Felt et al. 2013). This shift has arguably created problems for the knowledge base of science, casting doubt on the reliability of published results (Fanelli 2012; Prinz et al. 2011; Schatz 2014).

In these cited strong statements by eminent senior scientists, an anxiety surrounding what these quantitative metrics mean for younger generations of scientists is palpable. They warn that the current conditions will be “discouraging even the most outstanding prospective students from entering our profession” (Alberts et al. 2014: 5773) and worry about the impacts of the crisis on the work of those already engaged in life science careers. These senior scientists represent a generation that was socialized under different framework conditions; however, these scholars have also contributed to the development that they now criticize, and they partially continue to perpetuate it.

What do young researchers, those not in tenured and safe positions make of these criticisms? In late 2013, in line with the criticisms described above, a Nobel laureate announced that his laboratory would no longer send papers to *Nature*, *Cell* or *Science* because those journals were “damaging science.” In the comment section of a *Guardian* article reporting this story, a researcher responded as follows: “that’s the sort of stance you can take when you’ve got a Nobel Prize behind you. Us mere mortals, however, have to get publications in top journals if we wish to continue to be funded or employed.”^1^ As anecdotal evidence, this posting and the support it received speak to a generation gap in the sciences. This points to the importance of giving more attention to the positions of junior researchers on these issues.

This paper will focus on those who are currently growing into the academic life sciences: PhD students and postdocs. Considering these young researchers’ perspectives is important for the following reasons. First, they are particularly strongly affected by both hyper-competition and shifts in the ways in which scientific work is evaluated, as both dynamics are intrinsically linked to career structures and the processes of institutional staff selection. Second, considering the perspectives of young life science researchers also opens a window into current academic socialization in this field. Previous studies have highlighted the role of graduate studies in enculturating novices into their respective disciplinary cultures of knowledge production (Campbell 2003; Delamont and Atkinson 2001). Through this socialization, novices learn not only which questions to ask and how to produce knowledge but also how to recognize good research and how to live and work together as scientists. However, the existing literature on academic socialization focuses almost exclusively on graduate studies, neglecting the postdoc period, which has become an important early career phase (Åkerlind 2005; Müller 2014). Those with long-term perspectives in academia are selected well into this phase, not directly after the PhD. Furthermore, the literature does not consider how the valuation of researchers and their work has changed in institutions that are increasingly strongly governed by the logic of New Public Management (Burrows 2012; Shore 2008; Sparkes 2007). These changes are at the core of the perceived crisis in the life sciences, but they are also pervasive in many other research fields (Mills and Ratcliffe 2012; Stöckelová 2012). A better understanding of the interplay between how research is valued and how young researchers learn to live, work and produce knowledge in the sciences seems necessary—not least because those being socialized today will shape the cultures and practices in the sciences for decades to come.

In this paper, we will analyze how PhD students and postdocs in the Austrian life sciences ascribe worth to people, objects and practices when they discuss their present and future lives as researchers. Like many other higher education systems, Austrian academia has seen a strong shift in both career structures and discourses and practices aiming to define and assess the quality of research over the last decades, as well as considerable change in the governance of academic institutions. These changes are expected to have an effect on researchers’ valuation practices.

This paper will open with a section developing its theoretical background. Our conceptual approach builds on recent contributions in the field of valuation studies. This emerging field is interested not only in how actors draw on different evaluative principles in their practices but also in how valuations affect actors’ reflections about and decisions regarding these very practices. In this paper, we will analyze the different evaluative principles that young researchers draw on when discussing topics related to their current and future lives as researchers. To develop a deeper understanding of how young researchers refer to particular evaluative principles in a given context, we will develop the notion of “regimes of valuation,” which aims to trace how the evaluative principles that young researchers regularly learn to refer to in their socialization are grounded in discourses and institutions, building on particular moral and material infrastructures. After providing more details about our study and its context, we will develop our empirical argument. We are interested in how young researchers are socialized into different valuation practices at different stages of growing into the sciences. We will ask whether PhD students and postdocs draw on different evaluative principles when discussing a) their biographical futures, b) working together in research and c) the epistemic dimensions of their work. Our analysis will show that PhD students relate to a more diverse repertoire of evaluative principles than do postdocs, who base their decisions on the logic of one dominant regime of valuing research. In our conclusion, we will discuss the implications of this finding.

## Valuing and Doing Research

The new field of valuation studies is concerned with valuation as a social process, that is, with how the worth (both economic and social) of people, objects and practices is produced, assessed, distributed and negotiated (Kjellberg et al. 2013; Lamont 2012)^2^. Many contributions in this line of work share an interest in viewing valuation as practice and in examining how acts of valuation relate to actors’ practices and decisions.

This raises the issue of reactivity, which is how actors react to the fact that their practices are being evaluated and how they change their behaviors to improve their evaluation performance. Espeland and Sauder (2007) describe how academic institutions adapt their behavior to achieve better rankings. They show that the reflexive consideration of the evaluation process and the resulting re-shaping of institutional practices become an inextricable part of institutional work. Writers who comment on the impact of New Public Management on academic institutions have pointed to the multitude of metric assemblages that measure the conduct of individual researchers (Burrows 2012; Felt and Stöckelová 2009), the normative power that they exert (Stöckelová 2012), and the emotional burdens that they can cause (Sparkes 2007). Numerous studies have documented the impact that these new ways of measuring and defining research excellence have on collective actors, such as higher education institutions and scientific fields. They have found that considerable diversity exists in how these collective actors are affected by and react to these changes (Gläser et al. 2010; Paradeise and Thoenig 2013). However, little is known about how such changes affect different status groups within a field or institution. Only a few studies investigate how researchers relate to being evaluated (Linková 2014), how they engage in valuing their own work (Rushforth and de Rijcke 2015), and how this valuation shapes the way that they work and live as researchers and their decisions about whether to stay in or leave academia.

Studying the nexus between acts of (e)valuation and research practices raises the question of how to conceptualize the figures of valuation that actors in a specific domain (habitually) draw on to inform, orient or justify their actions. Valuation studies scholars have developed concepts to analyze how actors account for their valuations and their actions related to them. Boltanski and Thévenot (2006) have proposed six different *orders of worth* that societal actors refer to in justifying their actions, each with its own distinctive principles of evaluation, its own grammar of associating ideas and concepts and its own material tests for determining the worth of individuals or objects. They stress that actors’ capacities to reflexively relate to different orders of worth are acquired through socialization experiences in which they and their practices are evaluated according to specific orders of worth. Subsequent authors have taken up Boltanski and Thevenot’s interest in studying how actors refer to figures of valuation in their practices, but they have mostly found that their deductive scheme is too rigid for the empirical study of valuation as practice (Heuts and Mol 2013; Lamont 2012; Stark 2009). Rather than limiting their interest to how a fixed set of pre-defined orders of worth is referred to in social interactions, they have proposed empirically re-constructing the *evaluative principles* (Stark 2009) or *registers of valuation* (Heuts and Mol 2013) that are specific to particular empirical situations and studying situations in which it is uncertain which evaluative principle should be applied. In analyzing valuation practices in specific empirical settings, valuation studies scholars have distinguished between hierarchical and heterarchical constellations, depending on whether a specific situation is dominated by one specific evaluative principle or if a larger repertoire is available to the actors (Lamont 2012; Stark 2009). Heterarchical contexts are assumed to provide better conditions for social resilience because they offer a plurality of repertoires in which individual worth can legitimately be claimed. Studying high-tech companies, Stark (2009) argues that heterarchies also support intellectual innovation. His work shows that these companies thrive on ambiguity among coexisting evaluative principles at work, which creates uncertainty, enables entrepreneurial action and ultimately fosters organizational reflexivity.

Our approach in this paper shares these authors’ concerns to empirically re-construct rather than deduct patterns in how researchers ascribe value. Prior work has argued that researchers experience increasingly normative ways in which research in general and their own work and lives in particular are valued (Burrows 2012; Sparkes 2007). We will not limit our analytic gaze to the normative principles that are criticized in this literature or to young researchers’ reactions to them. Instead, we will attempt to map the breadth of different evaluative principles that young researchers employ to ascribe value to people, objects or actions when they discuss their current and future lives as researchers. We will define an evaluative principle as any logic or set of rules that our respondents explicitly or implicitly refer to when making a statement about worth in a particular situation. Our analytical work will focus on describing these evaluative principles, and on studying which group of young researchers (PhD students or postdocs) applies which principles in which situations. For this purpose, we find it important to consider that young researchers not only use evaluative principles to *evaluate -* that is, to assess the worth of their work and those of others. Researchers also employ evaluative principles in actions that aim to *valorize* (Heuts and Mol 2013; Vatin 2013) - that is, to build and increase the worth of their work and lives in the sciences. For example, when planning and conducting their next project, researchers consider the principles along which their work will be evaluated. Therefore, evaluative principles become important elements in how researchers think about and plan their biographical futures in and beyond science.

*Evaluative principles* will be the central focus of our empirical analysis. However, although speaking of *evaluative principles* or *registersof valuation* aptly stresses the situated and situational character of valuation practices, the evaluative principles used in acts of valuation also draw on wider moral/discursive and material infrastructures and institutionalized social practices of valuation (Kjellberg et al. 2013). Prime examples include the metric assemblages that pervade contemporary academia, the calculative infrastructures that they build on and institutions’ heterogeneous ways of using them (Burrows 2012). Therefore, we will use *regimes of valuation* as a concept that complements our empirical analysis of evaluative principles. Thus, in this paper, *evaluative principles* denote how worth is ascribed and argued for in a concrete situation, and *regimes of valuation* point to the broader discursive, material and institutional background this concrete evaluation draws on. We assume that regimes of valuation are comprised not only of institutionalized discourses, practices and material and digital infrastructures, but also of people living in, complying with and resisting these very regimes. These acts of compliance, resistance and debate make regimes dynamic, perhaps causing them to change^3^. In our understanding of the term, speaking of *regimes* sensitizes us to consider the material and discursive structures that make patterns of valuation durable (Kjellberg et al. 2013), but also to take into account actors’ own agency in relation to these structures^4^. Regimes of valuation suggest particular evaluative principles to actors who then apply, modify or reject them in concrete situations. Some regimes of valuation have more normative purchase on actors’ practices than others do, and they may thus restrict the actors’ capacities to act against the evaluative logic suggested by this dominant regime. A regime’s normative power may be expected to correspond with the degree of its institutionalization in discourses and practices. In analyzing our material and re-constructing regimes of valuation, we must look for traces of how our respondents ground the concrete valuations that they make and the principles that they use to do so in a broader narrative frame, which legitimizes this particular form of valuation.

## A Person-Centered Perspective on Cultures of Knowledge Production

This paper attempts to study young life science researchers’ cultures of knowledge production through the lens of valuation practices. In doing so, it utilizes a less comprehensive approach than classical studies of cultures of knowledge production, which aim to analyze the co-production of epistemic and social orders in entire fields of research (Knorr-Cetina 1999; Traweek 1988). This line of work has been surprisingly reluctant to engage with institutional and organizational changes and the different ways in which they affect specific cultures of knowledge production (Garforth and Kerr 2010). Additionally, it has contributed less to understanding how individual researchers navigate and cope with the complex realities of contemporary research landscapes. A more person-centered approach seems better suited to tackle this complexity.

In a study of contemporary knowledge cultures in academia, Felt (2009) recently suggested studying “epistemic living spaces” or researchers’ individual or collective perceptions of “the multi-dimensional structures—symbolic, social, intellectual, temporal and material—which mold, guide and delimit in more or less subtle ways researchers’ (inter)actions, what they aim to know, the degrees of agency they have and how they can produce knowledge” (ibid., 19). Focusing on the person as the agent of knowledge production, this concept aims to explore how ways of knowing in academia (i.e., epistemic approaches) are coproduced with ways of living in academia (i.e., the cultural specificities of particular research fields and geographic contexts). This concept allows for an analysis of how societal imaginaries and policy framings as well as changes in research assessment and monitoring exercises are experienced by individual researchers and how they affect their practices. From our perspective, regimes of valuation constitute one particularly crucial structure that molds practices in contemporary research.

Following this perspective, we have developed a specific methodological approach to capture the interrelations between the epistemic and social aspects of researchers’ epistemic living spaces. We call this approach the “reflexive peer-to-peer interview.” In line with recent work in very different traditions of qualitative research (Lynch 2000; Mills and Ratcliffe 2012; Plesner 2011; Stark 2009), we conceptualize reflexivity not only as a crucial virtue for social science researchers, but also as a capacity of social actors in general that constitutes a productive resource for qualitative studies of social phenomena. Our approach prompts our interlocutors to reflect on how the biographical, institutional and epistemic dimensions of the decisions they take related to their past, current and future careers are entangled. In this paper, our analysis will focus on respondents’ narrations of the role of valuations in their current practices and in their plans for the future. Due to the limited scope of a journal article, we cannot relate respondents’ valuation practices to their wider biographical backgrounds, and we will only bring in past experiences when they are crucial for analyzing present valuation practices.

The basic structure of the interview conversation follows that of a biographical or life story interview. However, rather than inviting only one linear narration, we used interviewer interventions to foster explicit reflections on the conditions that had shaped each individual’s perspectives, plans and decisions. These interventions were accomplished by (a) temporalizing, i.e., inviting the interviewees to relate past, present and future contexts and decisions to one another; (b) comparing, i.e., inviting the interviewees to compare their perspectives and arguments with those of other scientists who were relevant to them (e.g., a supervisor or a colleague who chose a very different career strategy); (c) perspectivizing, i.e., asking the interlocutors to retell particular aspects of their biographies from different viewpoints (e.g., adding a narration of the social context of their development to a story of seemingly purely epistemic choices) and (d) relating their experiences and practices to specific buzzwords in current science and science policy discourses, such as “mobility” or “competition.” Each of these strategies not only fosters reflection on a general level but also invites the interview partners to provide more explicit accounts of the evaluative principles related to their perspectives, actions and decisions. In this process, the interviewer plays a very active role (Holstein and Gubrium 1995) by attempting to induce the respondent to reflexively explore her or his story and self-presentation. In our experience, this process is aided by the shared role of the interviewer and the interviewee as academic peers, although this particular role constellation also introduces limitations that must be reflected in the analysis (e.g., that the interviewer and interviewee might tacitly share a particular definition of the quality of research).

Our analysis is based on interviews with 16 PhD students (9 female, 7 male) and 16 postdocs (8 female, 8 male). All respondents were employed in research groups at public universities and academies, which a project advisory board from the life science community had identified to be relevant research laboratories in a key subfield of the life sciences in Austria. These subfields included “red” (biomedical), “green” (plant) and “grey” (microbial) life sciences and bio-informatics. The interviews are part of a larger sample of 51 interviews in total, in which senior researchers were also interviewed. As the sampling process was based on the research groups, senior researchers, postdocs and PhD students were interviewed in each research group, which allowed us to better compare their perspectives, e.g., on living and working together in this specific group.

Approximately half the post-docs were born in Austria, while the other half were born elsewhere. In addition, seven of the 16 PhD students were not born in Austria, and they had come to Austria to earn their PhD. Whereas all interviewed PhD students were well into the second halves of their theses, the postdoc sample included respondents with quite different research experiences, ranging from researchers in their first postdoc year to others who were returning to Austria after two postdocs abroad. PhD students’ ages ranged from mid-20s to early 30s, with the highest proportion of PhD students being in their late 20s. Postdocs’ ages ranged from late 20s to early 40s, with most being in their early 30s^5^.

The interviews were conducted in English or German between 2007 and 2009. They lasted between 90 and 150 minutes and were recorded and fully transcribed. Informed consent was obtained before each interview. The informed consent form assured the participant of strict anonymity, which means that any details, such as a specific research field, university or nationality, that might make a person recognizable will be obscured in this analysis.

For the analysis of the material, the research team followed a grounded theory approach (Charmaz 2006), with multiple successive rounds of open coding. Questions about how to ascribe value to scientific practices, objects, institutions and people (including themselves) emerged as an interesting research theme during the analysis. Initially, our coding process did not focus on comparison of PhD students and postdocs; it instead sought to map young researchers’ statements about valuation practices. The major difference between PhD students and postdocs that this paper will report emerged as a result of the analysis.

## Contexts: Austrian and International Academic Labor Markets

Austria presents an interesting case for studying how researchers discuss the value of their work and lives in academia. In recent years, Austrian academia has seen a strong shift in both academic career structures and the discourses and practices that aim to define and measure the quality of research. Both in policy and within institutions, debates have focused on improving the quality of research by adhering more closely to what are framed as international standards, particularly in awarding research funds and in employing and promoting faculty. At the same time, there has been a shift away from the block funding of researchers and research organizations and toward competitive third-party funding and a focus on quantitative performance indicators, such as publication numbers and impact factors, in hiring practices. In this general effort to catch up with countries that are regarded as “innovation leaders,” specific research fields have received particular attention. The life sciences constitute a key area of Austrian specialization, and they have attracted considerable national financial support in the form of new research institutes and a dedicated government research program.

In addition, career structures have gradually but fundamentally changed since the mid-1990s. Traditional academic career trajectories, in which university faculty below the full professor level could achieve civil-servant status and hence secure a lifetime position by successfully concluding a *habilitation*, were repealed. For nearly a decade, no clear career trajectory existed within Austrian academic institutions, and all junior staff were employed with temporary contracts only. In 2009, a new collective bargaining agreement established a career trajectory that was similar to a tenure-track model, but which only led to the rank of associate professor. However, in a period of relative financial austerity, the number of these new positions remains low, and a strong discourse emphasizes the importance of a good balance between tenured and non-tenured staff. Formal evaluations of individual staff performance remain a rare exception beyond tenure track assessment procedures. Legally, individual performance should be discussed in formal annual appraisal meetings between staff and direct superior (group leader or head of department). However, if and how these meetings are conducted and if any binding agreements result from them varies widely between and within institutions. Often, they remain informal in character and may even be omitted particularly in project employment contexts.

Although this background is important for our study, our interviewees’ relationships to the Austrian context varied. Their main frame of reference was generally an internationalized academic job market rather than an Austrian one, even though the issue of being able to stay or to return to one’s home country was raised in our interviews. Among both PhD students and postdocs, nearly half of our interview partners had not completed their undergraduate studies in Austria and had come to Austria for specific research appointments, and many of the Austrian-born interviewees planned to take their next career steps abroad. At least in the European life sciences, the early stages of research careers do not typically take place within a national academic labor market and instead occur in the flows and markets constituted by international academic mobility.

## Empirical Findings

In the following empirical sections, we will analyze how PhD students and postdocs in the life sciences ascribe worth to people, objects and practices when they discuss their present and future lives in the academic life sciences. We will attempt to interpretatively reconstruct the evaluative principles and possibly the regimes of valuation to which they consider themselves subjected and explore how they relate them to their practices and decisions in three areas of academic work and life that emerged as central topics in our interviews:

- working together in research;
- epistemic practices and decisions; and
- biographical options and considerations.

In the following two sections, we will discuss the accounts of PhD students and postdocs. As we will show, the framing and importance of these topics varies considerably between these two groups. Hence, we will not analyze the topics in a fixed order in either section; we will instead begin with the topic that was most relevant to the narrations of each group. As a brief addendum to the empirical section, we will then discuss both PhD students’ and postdocs’ critical reflections on the regimes of valuation that are central to their practices.

## Evaluative Principles and Regimes of Valuation in PhD Students’ Accounts of Living and Working in Research

### Working Together in Research

What makes a good research group? This question was very important for most of the PhD students we interviewed, either when discussing their decision to do a PhD in a particular group, when describing their current work context and their satisfaction with it, or when thinking about their future as researchers. What were the evaluative principles they used to determine whether a group is a “good” one to work in? When discussing their initial decision to do a PhD, or their current work, PhD students reported that the publication output of the group or its international reputation were not necessarily the primary criteria for determining the value of a group. These criteria were mentioned, particularly when PhD students considered the value of their group for their future academic careers. But in describing their current practices, it was often more important if the group provided an intellectual and social climate that allowed the PhD student to develop through working together in research. PhD students often used terms that have domestic connotations, such as “*family*,” “*home*” or “*nest*,” to refer to their groups. For example, a male PhD student referred to his group as “*small and family-like […]*, *where no one is just struggling along on their own*” (PhD15m^6^). As the metaphor of the family suggests, students expressed moral expectations not only for themselves but also towards other members of the group, which centered on the individual’s contribution to the collective effort. A female Austrian PhD student phrased this collaborative impetus as follows:

Data that I have I know will be important for someone else, I share it. I have never ever had a problem of going to someone in the lab and saying, “I need your help,” or, “I need guidance on this stuff. Do you have any suggestions?” The person might say, “I have something to do now, but come to me in half an hour or come to me tomorrow.” This might happen, that the person doesn’t have time at the moment. But I never had anybody in the lab say, “I will not help you,” or, “I don’t want to do it,” or something like that. Never^7^. (PhD4f)

In our interpretation of this quotation, and many similar ones in our material, we see the general evaluative principle PhD students stressed for research groups enacted in how they value the behavior of colleagues in everyday interactions. Here, the colleague does not have to stop his/her individual projects immediately to help, but he/she is expected to demonstrate a general willingness to help in the near future. The worth that PhD students assigned to individuals and their activities was predominantly based on their intellectual sociability and willingness to support others. These qualities were ascribed and tested in a multitude of everyday interactions. Offering support in managing the practical everyday problems of doing research was considered appropriate and valuable behavior, whereas reluctance to give advice, to contribute one’s share to managing common resources or to offer emotional support in times of frustration was not.

Group leaders, as the heads of their respective “*families*,” have a particular role in this context. They are expected to manage “*the politics*” of academic work and research funding and to generate a protected space in which PhD students can concentrate on the experimental aspect of scientific work^8^. From the PhD students’ perspectives, group leaders are also obligated to care for their group members. In addition to general intellectual sociability, this obligation particularly implies that group leaders ensure that their students “*finish well*.” The supervisor is responsible for managing and mitigating the epistemic risks^9^ in his/her students’ projects, for instance, by assigning safe backup projects that allow students, whose primary lines of experimentation fail, to still complete their PhD.

We interpret the dense use of related evaluative principles in our PhD students’ discussions of their work in the laboratory as indicative of the presence of a regime of valuation centered on learning, mutual care and collaborative work as the key principles for ascribing worth to individuals, groups and actions. Discursively, PhD students first often implicitly grounded their valuations in this regime on a narrative of the PhD as a learning phase. Second, they regarded the rights and responsibilities related to caring and learning as institutionalized in the hierarchical structure of the life science research group. Group leaders are expected to care for all group members; postdocs are expected to support PhD students; and more senior PhD students are expected to help junior PhD students. Third, additional important discursive reference points that legitimized this regime and its evaluative principles were stories and urban legends. Re-told in many different interviews, though always as rumors about unnamed research groups elsewhere, particularly “*in America*,” these stories demonstrated the principles of the regime by deploring their violation. In one of these stories, group leaders assigned multiple researchers the same risky topic, stirring competition instead of cooperation as a means of increasing the overall chances of success. Such group leaders were perceived to negate their responsibilities of caring for each of their PhD students equally. In other anecdotes, group leaders were implicitly criticized for allowing the “*geniuses*” in their laboratory to contribute less to maintaining common resources because they contributed more to the laboratory’s performance through their individual productivity. One female PhD student recounted an instance in which a female visiting professor told her and other female students to be careful to not take on too many group-related responsibilities and end up as the “*cleaning woman of the lab*” and to ensure that they spent more time working on their own productivity and careers.

This latter story hints at a latent conflict between the regime of valuation based on care and collectivity we have described thus far and another regime focusing on individual productivity we will encounter in more detail particularly in our analysis of postdocs’ statements. The tension does not necessarily lie between care and productivity per se, as collective productivity may be aligned with the principles of the care regime of valuation. Consider how a PhD student describes this positive relationship between productivity and care:

It’s important to not only pay attention to yourself and your own work but also to the group. Because, of course, it’s important that your lab has a good reputation and that everybody makes progress and contributes their share. (PhD3f)

In this quotation, caring for others and helping their progress benefits everyone because it increases the quality of the collective work and hence the reputation of the laboratory, which may rub off on individuals as they apply for postdoc positions in other laboratories. However, in the “*cleaning woman*” story above, individuals are portrayed as harming or improving their career chances by focusing on individually ascribable productivity rather than contributing to collective aspects of work. As we will see in the next section, PhD students tended to focus more on individual productivity as an evaluative principle for their work when they discussed their future careers, particularly in academia.

### Biographical Options and Considerations

By definition, the PhD is a temporary phase—and hence PhD students’ plans for the future were an important topic in our interviews. Very few of the PhD students we interviewed considered themselves committed to clear future career trajectories. For many, academia seemed the most interesting career choice; others favored industry; and some considered the completion of their PhD and perhaps even a postdoc as a way of keeping their life options open.

The specific evaluative principles that PhD students referred to in their accounts differed according to the careers that they favored and the regimes of valuation that they assumed were institutionalized in the labor markets of the areas into which they planned to move. As such, the most pressing valuation question was how they and their work would be valued in these biographical transitions. In one way or another, all described their lives and work as acquiring worth by efficiently producing individually ascribable products or properties, which would serve as assets in a specific market of future options.

Not surprisingly, students who aimed to pursue academic careers stressed publications as the most important product. Consider the following quotation:

So, that’s what I know to be true for people who would like to stay in research and also for me […] I would say the main aim of doing the PhD is getting publications (laughs). That’s what it’s about. (PhD16f)

In this quotation, when the student discusses a future career, the discourses about learning and care that we encountered above in the PhD students’ accounts of their everyday work have been replaced by a vocabulary of individualized work and productivity. However, the student immediately continues as follows:

So, of course, it’s not only about that but also about adding to the knowledge base. But, in the end, the publication is the product of the work, one might nearly say. (PhD16f)

In our interpretation, this second part of the quotation points to a conflict between the two evaluative principles that the student perceives: a principle that values people as part of a larger collective endeavor and one that only focuses on individual productivity. Although reconciling the two may seem possible in this quotation, we will find more explicit statements on the tensions between them in our analysis of the postdocs’ critical perspectives.

PhD students who were leaning toward careers outside of academia had different perceptions of publications and their value for their future careers:

As I’ve heard, when you go for a career on the industry side, you shouldn’t publish too much. Because they don’t like that, and they might say you have worked too much and had too little time for social contacts. (PhD9f)

Here, the students’ assumptions about the evaluative principles used in industry employment lead her to be skeptical about producing too much in terms of publications. Sociality is assumed to matter in research in the private sector—signaling a continuity or at least compatibility with the regime of care that we have sketched in the previous section.

Because some signs of productivity that are valued in one future work context might be viewed with skepticism in others, students who had not yet decided on a specific career path were most interested in acquiring properties that would signify worth across the different regimes of valuation that were specific to particular work contexts. In our respondents’ accounts, a broad knowledge of different research methods was the best example of this approach, as it was considered recognizable across different institutional contexts, such as academia, the pharmaceutical industry and biotech startup companies. Within academia, it was also considered to broaden the individual’s career options in terms of possible research fields.

### Epistemic Practices and Decisions

Which evaluative principles did PhD students refer to when speaking about their considerations and choices in their epistemic work? Generally, PhD students’ topics in the life sciences are assigned by their supervisor, limiting the range of epistemic and methodological choices that they can make. However, we found a wealth of stories about choice—about students who chose the group in which they wanted to work and thus a specific approach or about students who chose a project out of the options offered by the group leader. Within these stories, three main lines that determine a topic’s worth can be distinguished. The first applies individual fascination and motivation as an evaluative principle; the chosen topic is “*cool*” or “*fascinating*.” The second stresses the importance of choosing a project that, in the long run, will contribute to the common good by being “*close to humans*” or relevant for “*curing diseases*” or by having medium-term applications. The third concerns the topic’s productivity in terms of the methods that it allows the student to learn, which includes both a fascination with attempting different approaches and methods as assets that can increase a person’s worth in job markets. Interestingly, PhD students’ accounts contain very few hints of any conflict between these three lines that establish the worth of their epistemic work. In addition, their expected productivity in terms of publications is not mentioned as an evaluative principle when choosing a project or when making epistemic decisions in the course of an ongoing thesis project. On the contrary, a number of students mentioned choosing epistemically risky projects despite the dangers that they might pose for their publication record. Consider the following quotations:

As a PhD student, [a risky topic that fails to deliver results] doesn’t have too many consequences. If there are good results, then it’s great. If not, well, then I [publish] something good later. I think later [in an academic career], you need to be more grown up in that respect and assess which risks you should take and which you shouldn’t. And you also will be. […] I think the further you move up the career ladder, the more you have to stay away from risk. (PhD2m)

There are these [topics] where you’re nearly guaranteed to finish in three years, but you also know that you will not learn more than three or four methods in this time. […] But I didn’t want that. I wanted something that maybe is a bit scary to begin with because you have no guarantee when you will finish, what the outcome will be, and whether it will be interesting to anybody. But, in the end, this is about the biological context and whether this will be relevant for humans. That’s what I wanted to do. And, in doing so, I’ll learn far more methods. (Phd3f)

The first quotation stresses that the PhD is a time to take epistemic risks—not least because this risk taking happens in the relatively protected learning space described above, in which the student, as a maturing “child,” is allowed to experiment and make mistakes. The second quotation relates the choice of a risky topic more to the different evaluative principles in which a PhD students’ work can acquire worth. Even if a project fails to deliver publications, it might still be useful in terms of learning different methods, which can be assets in acquiring a postdoc position or a job in the private sector; the work might be deeply fascinating, or it might be a first step in addressing wider problems of humanity. Additionally, performing well in the regime of care and collectivity in the laboratory will likely lead to being assigned a backup project to complete the PhD nonetheless; and a favorable recommendation by one’s supervisor might compensate for a suboptimal publication output. This range of possible evaluative principles, in which a project that fails to generate publication output can still have worth, allows for risky choices and lends resilience to PhD students’ biographic and epistemic pathways.

The evaluative principles that we have observed in our PhD student interviews can be interpretatively related to regimes of valuation in different ways. In the first section on working together, we have observed relatively dense traces of a regime that centers on intellectual sociability, care and collectivity, its moral prescriptions and its institutionalization. Other evaluative principles, including those focused on publication output, seem institutionalized in students’ perceptions of future labor markets. But generally students referred to these principles and described their institutionalization less frequently, which may indicate that the regimes in which these evaluative principles may be institutionalized are less relevant for PhD students. A third group of evaluative principles, including individual fascination or the relevance of research for human health, largely stood alone, only loosely related to general discourses about the nature and sense of academic work. These evaluative principles were often related to the biographical reasons why students had chosen to do a PhD and to pursue a certain topic, but students did not describe any institutionalization of these principles regarding their current or future practices. This is a particularly interesting finding with regard to the wider societal relevance of research, which is an important topic both in the media and in discourses about research in Austria within institutions of higher education. However, these discourses do not seem to translate into institutionally grounded evaluative principles that make a difference in researchers’ practices.

## Evaluative Principles and Regimes of Valuation in Postdocs’ Accounts of Living and Working in Research

### Biographical Options and Considerations

In contrast to the PhD students’ accounts, the dynamics of everyday work were not the main topic for the postdocs we interviewed. Instead, they were deeply concerned with their futures and how their research would contribute to their achievement of career goals. Postdocs did not discuss the possibility of different career trajectories. For them, deciding to do a postdoc was the equivalent of choosing to pursue an academic career. Alternative careers were viewed as a product of failure and thus were hardly considered.

Nearly all the interviewed postdocs shared a preoccupation with—and somewhat of an anxiety about—their career prospects. They were deeply concerned about the worth of their research work and their worth in the scientific labor market, as this postdoc explains:

The (…) higher you are in this hierarchy, the more competition you can feel. […] Because a lot of postdocs have to fight for the best publications to get a group leader position in the future. […] We fight for money […], and of course we fight […] for being first, you know, because only if you are first to publish then it’s cited a lot. […]. And this is somehow a measure of success in science—the number of citations. (PDoc1m)

In this quotation, individuals’ worth is defined by their ability to succeed in competition, based on their productivity in acquiring tokens of academic quality that may count for something in the international academic labor market, that is, indexed publications, grant money and recorded citations.

Why did postdocs focus so much on individual productivity? Knowing that only a very small proportion of them will be able to become group leaders and stay in research, they considered themselves engaged in a fierce competition. This experience of competition is augmented by the spatial and temporal organization of the postdoc phase. Postdocs are expected to be mobile, that is, to change research groups and, better yet, countries after each postdoctoral appointment of typically two to three years. The interviewees considered two to three postdocs to be the norm for those pursuing scientific careers; after this postdoc phase, they can possibly settle into a more long-term position.

The necessity of moving on to a new work context requires that postdocs prove their worth in ways that are transferable and standardized rather than tied to a specific local context. For early postdoctoral appointments, recommendation letters can transform locally proven worth, such as intellectual sociability or experimental skills, into a transferable currency. However, the relevance of recommendation letters was seen to strongly decline as one started to enter the competition for group leader positions.

As the reputation of the groups and places associated with postdoctoral appointments matters, every transition from one appointment to the next is regarded as connected to a test of the worth of one’s past and future research capacity compared with that of others. Consider how the postdoc below justifies her work at internationally renowned universities as an important part of her scientific vita:

So, I think this postdoc abroad is extremely important for the career and what your CV says about it. […] Because, there, you really have to work on your own and show what one is capable of; that’s why it is so decisive. If you prevail against the enormous competition at such a top university, […] that says a lot. (PDoc3f)

Interestingly, in this quotation, neither the general reputation of the university nor the epistemic opportunities that it might offer for this specific postdoc’s work are cited as central. Instead, the competitiveness of the place and the individual’s ability to survive in this environment constitute worth^10^. Very different from PhD students, postdocs view themselves as individuals competing on a global market rather than as part of a specific localized research collective^11^.

In postdocs’ biographical considerations, we re-encounter a basic evaluative principle to which the PhD students also referred—determining an individual’s worth through his/her productivity. Compared with our PhD students, the postdocs regard this evaluative principle as far more central, and they describe it as institutionalized in a number of discourses and evaluation infrastructures: in their perception of the dynamics of the international labor market and the hiring conditions for group leader positions, in the temporal and spatial structure of the postdoc as a succession of time-limited mobility episodes, in a general discourse about the importance of excellence and competition, and in the material forms and formats of counting and accounting for research output. Other than for PhD students, for postdocs it is this regime of valuation that generates a strong moral imperative. From the perspective of the individual postdoc, knowing which performance will be needed to succeed in competition seems impossible because, on a global scale, the competitors are too numerous and their performances cannot be monitored. Hence, maximizing their performance in line with the rules of this regime of valuation seems imperative to many postdocs.

Therefore, postdocs aim to make biographical decisions that maximize their individual productivity and competitive performance. These decisions do not only impact their professional practice, but also strongly affect their private lives. They discuss “*logging out of life*” for the postdoctoral period to “*show that one really belongs in science*,” “*camping in the lab*,” and postponing family plans. The readiness to sacrifice other biographical plans is viewed as an indicator of a person’s worth in a discourse that assumes that only those with total dedication can become successful researchers. Other biographical aims are postponed until later phases, assuming that these phases involve less competition; however, quite a large number of respondents expressed doubts about whether such phases were possible in contemporary academia, even if they succeeded in becoming group leaders.

In postdocs’ biographical considerations, one regime of valuation was dominant. Other evaluative principles that were cited as ends in themselves in our PhD interviews, such as inspirational motivation, were referred to as resources in discussions of the postdoc phase. For example, epistemic fascination was not discussed so much as a value in itself but as a resource that was necessary for coping with the long working hours required to prevail in the competition.

### Living and Working Together in Science

Very different from PhD students, postdocs framed their relationships within the research group in terms of how the group aided their productivity and performance in the regime of valuation they saw as dominant. For example, one of our informants discussed his current project and the fact that if it would deliver promising results was unclear. He considered leaving the group if the project did not turn out to be productive, justifying this decision as follows:

I am sorry; it is kind of business. As a postdoc, you cannot stay too long in a place where you don’t get a kind of profit, you know, in the form of publications or good scientific data because it’s very bad for your future career and getting your own group and stuff like that. So… (PDoc1m)

Notice how our informant uses the introductory phrase “it is kind of business” to justify why the potential consequences of his decision for his future individual productivity weigh more heavily than any moral obligations that he might have to the group leader or his colleagues in his current group. As opposed to the accounts of our PhD informants, the central evaluative principle that determines the quality of a specific local context is not how well it enables learning and mutual support but how well it supports the postdoc in acquiring the credentials needed to further his academic career.

Of course, postdocs’ emphasis on competitiveness was not always as strong as in the quotation above. In discussing their everyday work, many still referred to the importance of good working relationships in the group. However, the ways in which they defined their rights and obligations toward other group members were very different from those of our PhD student informants. Teaching, learning and contributing to common resources played a role for postdocs, but these actions were not seen as valuable in themselves; instead, they needed to be scrutinized in terms of the extent to which they contribute to or inhibit individual productivity:

So, yeah, I mean, I think certainly […] whenever you ask anybody for help, there is always a question of, well, you know, maybe it’s not the first question that comes to mind, but eventually it’s a question of, well, am I gonna be on the paper, am I not gonna be on the paper. (PDoc9m)

As the quotation above illustrates, for postdocs, asking for help must be weighed against the risk of having to share authorship, and providing assistance should result in co-authorship on papers that result from the project of the person who was helped^12^. As such, nearly every act of technical or epistemic support constitutes an implicit exchange relationship; publication credits are received for the time and knowledge invested.

Postdocs’ individualistic and instrumental framing of their social relations in the laboratory stands in latent conflict with the hierarchical structure of research groups and the regime of valuation associated with it that our PhD informants described. Postdocs felt the need to prove themselves as independent individuals and to grow out of this hierarchy as quickly as possible; however, they still had to contend with the expectations that others had for them as group members. Consider the following quotation in which a postdoc talks about her ambivalent relationship with her lab leader:

Because if I sit here until I’m 40, […] when I apply for a grant, they will ask me why I didn’t become independent earlier. Then everything I do would be his [her current lab leader’s], you know. So, like, it was not my idea; it was his idea. It was not my project; it was his project. (PDoc2f)

### Epistemic Practices and Decisions

Assessing actions in terms of their expected future profitability in the dominant regime of valuation also influences how postdocs discuss planning projects and even choosing questions and model organisms. Consider the following quotation by a senior postdoc:

The higher you rise on the career ladder, the more the pressure rises when you […] choose a project. There are interesting projects, but you know it will be hard to find funding for them. Because if the reviewers assessing it say: This is interesting, but it is just not en vogue at the moment. Then it will also be hard to publish that. So, you really start planning at the very beginning [of the project]—what will I be able to write in a paper, which experiments do I need, and so on. It sounds much more calculating than you would assume it to be when you start out naïvely into a research career. (PDoc6f)

Here, the “calculating” mindset involved in epistemic decisions is juxtaposed with the “naïve” assumption that epistemic interests and considerations alone determine the choice and planning of projects. Of course, postdocs also speak about choosing topics that fascinate them. However, the space in which these fascinations can unfold is a priori structured and delineated by considerations of their expected productivity. This does not only apply to the planning of projects but also to how they choose methods and model organisms. Consider how a postdoc builds a complex argument about the different payoffs and risks of two methods in the context of the temporalities of contemporary academic careers:

So, everyone [working with mice as a model system] has to realize there is some risk involved. If I do cell culture and I have a well-defined question […], you look at that, there are results […] and you can publish that. […] On the other hand, if you have a [transgenic] mouse, that can mean that in two months you can learn as much as you can in five years doing cell culture. But considering that your contract [for a university assistant position] is limited to four years [at this university], and you need two years until you’ve got the mouse. And you might have to do some teaching as well. Then you might manage to do, say, one publication. And then you have to go. (PDoc7m)

Consequently, attempting to build a career using transgenic mice as the main model system is discussed as a “*risky game*,” as “*poker*,” or perhaps even as “*naïve*.” Interestingly, the risk involved in choosing topics, methodological approaches or model organisms is conceptualized very differently here from the way that it was in our PhD student interlocutors’ statements. For postdocs, epistemic risk is defined through the relationships between the expected data quality (ideally expressed through the impact factor level of the journal that accepts one’s publication^13^), the expected time needed to produce these results and the perceived likelihood that a particular project will fail. Within this equation, different risk management strategies are possible, from mixing high- and low-risk approaches to “*doing one high-risk project*, *and if that doesn’t work*, *then they say*, *OK*, *I’m out*” (PDoc6f). Epistemic risk is nearly synonymous with career risk, as an unwise epistemic investment is seen as almost inevitably leading to losing out in international competition.

Postdocs’ strategies for dealing with risk were very different from those of PhD students. PhD students were in a more resilient position to take epistemic risks because, from their perspectives, if their work failed to generate worth along one evaluative principle—such as individual productivity—they could compensate for this failure with success in others. For postdocs, only a narrow set of evaluative principle centered on productivity seemed to truly matter. Failure to produce results that can be transformed into publications or grant money cannot be compensated, and equals career failure. Whereas PhD students operate in a more heterarchical context and are able to take advantage of this context by taking epistemic risks, postdocs conduct their research in a context structured by one extremely dominant regime of valuation and hence make more conservative decisions.

## How PhD Students and Postdocs Critically Reflected on Principles and Regimes of Valuation

Much of our analytical work in this paper has focused on how our respondents implicitly referred to evaluative principles in discussing important aspects of their work and lives as researchers. In this section, we will briefly discuss whether and how both PhD students and postdocs explicitly and critically reflected on how evaluative principles structured their practices.

As long as they were discussing their current practices, PhD students hardly explicitly addressed the evaluative principles to which they referred. However, when they were prospectively discussing the option of staying in academia and doing a postdoc, many PhD students quite critically commented on what they considered the dominant regime of valuation in the postdoc phase and beyond. However, they did not aim their criticism at how worth is ascribed in the postdoc; they instead discussed the consequences of the dominant regime of valuation for their potential biographies. Consider these two quotations, which exemplify this position:

So, the fewest among the PhD students I know plan an academic career, and those who do, they’re really extremely good. Studied in no time, went to Cambridge and now have a postdoc at the Rockefeller University in New York. And their apartment is paid for by the university, and they have their *Science* and *Cell* papers. […] But as a mediocre PhD…, I mean I would be able to do that, but I simply don’t have the drive and am interested in too many other things. (PhD15m)

I don’t necessarily want to become a group leader. I also am not going to stay in science. Because if you look at the group leaders now, they have no functioning relationships, or they are in the lab night and day, or if they do have a family, they are completely stressed out […] You have to move somewhere else every four or five years and if you have private plans, that just won’t work. […] (PhD10f)

Indeed, a considerable number of PhD students we interviewed, more often women than men (even though the numbers are too low to make any broader statement), planned to opt against a career in research because they saw themselves as unable to (or simply did not want to) engage in this extreme competition and the biographical sacrifices that it demands.

Relatively few of the postdocs we interviewed explicitly reflected on the dominant regime of valuation that they were experiencing and its consequences for the epistemic and social dynamics of science. Those who did were largely critical of its consequences. A main point of criticism highlighted the loss of epistemic depth:

I was visiting a department in England and was nearly shocked to see how science there basically just aims at getting something written down and out the door. So it’s not about understanding what one is actually doing. (PDoc8m)

Others criticized the current focus on individual productivity and its accompanying devaluation of work invested in intellectual and social reproduction in the laboratory, which they believed would have negative consequences for the social organization of science in the long term:

So, impact. In the scientific world, that’s the only thing that counts, unfortunately. […]. But then other people do top work in supervising people, making sure that the lab runs smoothly, but maybe they get to do less research. Now they aren’t first or last author, but they are just as important. […] So, impact factors count when you apply for a professorship. […] But the person does not. But if you work with so many people [as a professor], you would also expect leadership qualities. (PDoc7m)

Essentially, critical statements such as this one argued for a broadening of the evaluative principles that are used to evaluate scientists and scientific work. However, in the context of our entire interview sample, we must add that these types of reflections were surprisingly limited. Most of our interviewee partners discussed their struggles to perform and survive in the current regime of valuation, but only a few reflected on the implications for science as both an intellectual and a social endeavor. Those few who did often struggled to find the words to express their criticism. First, they meticulously sought to avoid being viewed as buying into a certain nepotism and parochialism that was considered characteristic of the dynamics of careers in academia in Austria in earlier generations or as being afraid of “healthy” international competition. Second, they lacked any imagination regarding other potential regimes of research valuation beyond the currently dominant regime and its de-legitimized historical predecessors. Notably, our respondents only referred to past alternative regimes of valuation, but they did not imagine future alternatives to the currently dominant regime that they criticized.

## Discussion and Conclusion

This paper has offered some analytic insights into the current epistemic and social crisis of the academic life sciences (Alberts et al. 2014) through the perspectives of doctoral and postdoctoral researchers working in Austria. Its central observation is a stark narrowing down of the repertoire of evaluative principles that are available to researchers to conceptualize and prove the worth of their work as they advance in academic socialization.

Our empirical analysis has shown that life science PhD students refer to a range of different evaluative principles when discussing their current and future lives in research. This plurality of evaluative principles leads us to characterize their epistemic living spaces as heterarchical. As a prime example, we have described how this plurality enables PhD students to engage in risky epistemic questions.

By contrast, postdocs’ narratives mainly refer to one regime of valuation in which the worth of individuals is defined by their ability to succeed in competition based on productivity in terms of acquiring internationally accepted and transferable tokens of academic quality, that is, indexed publications, grant money and recorded citations. Other evaluative principles were hardly mentioned, and if they were, they were mostly discussed either in instrumental relation to this dominant regime or as obstacles in conflict with this regime. Postdocs’ epistemic living spaces may thus be described as being structured by a strongly hierarchical form of attributing worth. Virtually all decisions—epistemic, career-related or even private—were scrutinized in relation to their potential effects on the postdocs’ individual productivity and thus their chances of obtaining future faculty positions. Epistemically, this led them to potentially make rather conservative choices, avoiding unorthodox, risky or lengthy projects.

In growing into the life sciences as an academic field, young researchers are hence socialized into an ever-narrower regime of valuing their work and that of others. Those who choose to remain in academia move from a heterarchical epistemic living space into a hierarchical environment.

Both PhD students and postdocs did not see themselves as having significant agency to critically engage with the dominant regime of valuation. While PhD students located their agency in choosing between compliance and exit, most postdocs tended to treat the dominant regime of valuation as a quasi-natural order without alternative. The postdocs who did criticize the negative effects of this dominant regime lacked imagination with regard to potential changes—they saw no alternative regimes to which they could legitimately refer.

Our findings may be read to connect with broader debates about the re-structuring of academic work along the neoliberal agenda of the New Public Management (Shore 2008; Sparkes 2007). The increasing metric measurement of research performance, the temporalization of research work and the emphasis on competition are key elements of the dominant regime of valuation that we have analyzed for the postdoc period. Prior literature has pointed to the fact that the actual impact of changes in the governance of research on specific institutions or fields of research is often not as linear as might be assumed at first glance (Gläser et al. 2010; Paradeise and Thoenig 2013). With this caveat in mind, we would like to propose five hypotheses on the potential wider problematic implications of the narrowing of evaluative repertoires that we have analyzed in this paper—for science as both a social and epistemic endeavor.

First, the valuation dynamics that we have observed may create deeply unsustainable biographies for those opting for a career in research. Researchers’ intense focus on a distinctly inner-academic system of valuing research potentially keeps them from acquiring skills that might be valuable in other employment contexts. Additionally, to succeed in competing in the dominant regime of valuation in the postdoctoral period, they sacrifice other aims that have worth for them in their wider biographic plans, i.e., personal fulfillment not only in their epistemic and professional lives but also in their private lives. Such sacrifices may be expected to have dire consequences for the predictably high percentage of postdocs who will not remain in academia in the long term (European Science Foundation 2015).

Second, for the research system, the narrowing of evaluative repertoires could create problematic dynamics of selection and self-selection. Many of the doctoral students we interviewed planned to opt out of an academic career because they did not want to work and live like their postdoc colleagues. Research, at least in the life sciences, could indeed be becoming less attractive for young people whose biographic imaginations have no fit with the dominant regime of valuation. In addition to existing selection effects, such self-selection processes may further challenge the aim of fostering diversity in the scientific workforce. Particularly the role of gender, class and ethnic background merits deeper exploration than we could provide in this paper.

Third, the highly individualistic dynamics fostered by the current regime of valuation, at least for postdocs, are likely to challenge the social structures of the research system. The cohesion of local research groups and departments is likely to suffer as individuals are structurally encouraged to place their individual interests above the work needed to build and maintain collective intellectual structures.

Fourth, the narrowing down to one regime of valuation also may segregate academic science from other societal values and concerns. As our material shows, care for the solution of societal problems is still an important issue for PhD students, but it gradually loses importance in the environment in which postdocs work. Other than currently demanded by policy discourses and academic commentators alike (Felt et al. 2013; Stilgoe et al. 2013), academic socialization in the life sciences seems to structurally inhibit engaging with societal responsibility rather than fostering it.

Fifth, research driven by productivity concerns rather than other values also runs the risk of hampering its epistemic development. On the one hand, the conservative approach induced by the synonymy of epistemic and career risks (Sigl 2015) is likely to lead to a mainstreaming of research, to researchers choosing safe topics rather than pursuing risky intellectual breakthroughs and to a systematic under-reporting of negative results (Fanelli 2012). On the other hand, the need to stand out in a crowd may lead to deviant behavior, such as the premature publication of results, the exaggeration of the significance of findings, or even the fraudulent fabrication of data.

Further research is needed to explore the salience of these hypotheses. In identifying directions for future studies, reflecting the context of our case and its potential limitations may be useful. Our study focused on the life sciences because they have been at the center of research policy imaginaries in recent decades, not only providing them with ample funding but also exposing them to new governance logics. We argue that this specific constellation makes the life science a good case for observing general trends; however, studies are needed to explore the dynamics in other fields, which will likely be different. An additional important context in our study is Austria, with its strong impetus to internationalize research careers and to distance itself from a more parochial past. Again, comparative work with other nations seems necessary to better grasp the respective importance of local governance and global standardization processes (Paradeise and Thoenig 2013). Institutionally, our sample focused on well performing research groups with clear international perspectives. Studies that analyze institutions and groups with different profiles may reveal different dynamics.

This paper has also aimed to offer new theoretical tools for future studies of academic valuation practices. Our approach joins recent valuation studies in stressing the importance of empirically re-constructing the situated use of specific evaluative principles. However, it also stresses the importance of considering the institutionalization of these principles in wider regimes of valuation and the structuring and even coercive effects that they may exert on individuals’ valuation practices. In offering these tools, this paper aims to contribute to the wider agenda of research on academic valuation practices and their effects on the relationship between science and society.

## Footnotes

1 http://www.theguardian.com/science/2013/dec/09/nobel-winner-boycott-science-journals, retrieved 02.17.2015.

2 Of course, the question of what constitutes worth and capital in academic practice has a much longer tradition in the sociology of science and science and technology studies; see, for example, Bourdieu (1975), or Latour and Woolgar (1979).

3 The San Francisco Declaration on Research Assessment (DORA) is a very good example of this dynamic.

4 See Hecht (2001), for a similar use of *regime* in a very different context.

5 The gender distribution in the different subgroups mentioned roughly corresponded to the overall distribution.

6 In our respondents’ anonymized identifiers, PhD denotes PhD students and PDoc postdoc respondents. m (male) and f (female) denote the sex of the respondent.

7 Quotations from interviews conducted in German were translated by the authors.

8 See Davies and Horst (2015) for a more detailed account on the role of care in principal investigators’ management of their groups.

9 Our use of the term (epistemic) risk relates to efforts to manage the inherent possibility that research endeavors might not yield relevant results, even if they are conducted with high scientific competence.

10 Our postdoc informants’ mental maps of “top” and “competitive” places followed a stereotypical tacit geography (Felt and Stöckelová 2009), with American Ivy League institutions and a small number of European universities on top and the rest of the world as essentially *terra incognita.*

11 See Müller (2014) for a more detailed account on the individualizing nature of the postdoc period.

12 See Müller (2014) for a more detailed discussion of this point.

13 See Rushforth and de Rijcke (2015) for a similar argument.
